# *N*′-(5-Bromo-2-methoxy­benzyl­idene)-4-hydroxy­benzohydrazide methanol solvate

**DOI:** 10.1107/S1600536809022983

**Published:** 2009-06-20

**Authors:** Xue-Song Lin, Ya-Li Sang

**Affiliations:** a Department of Chemistry, Chifeng University, Chifeng 024001, People’s Republic of China

## Abstract

In the title hydrazone compound, C_15_H_13_BrN_2_O_3_·CH_3_OH, the methanol solvate is linked to the benzohydrazide molecule through O—H⋯N and O—H⋯O hydrogen bonds. The benzohydrazide mol­ecule adopts an *E* configuration about the C=N double bond. The mol­ecule is twisted, with a dihedral angle between the two substituted benzene rings of 35.7 (2)°. In the crystal structure, mol­ecules are linked through inter­molecular N—H⋯O and O—H⋯O hydrogen bonds, forming layers parallel to the *ac* plane.

## Related literature

For the biological properties of the hydrazone compounds, see: Khattab (2005 ▶); Küçükgüzel *et al.* (2003 ▶); Çukurovalı *et al.* (2006 ▶). For the structures of hydrazone derivatives, see: Fun *et al.* (2008 ▶); Wei *et al.* (2009 ▶); Khaledi *et al.* (2008 ▶); Yang *et al.* (2008 ▶). For reference structural data, see: Allen *et al.* (1987 ▶).
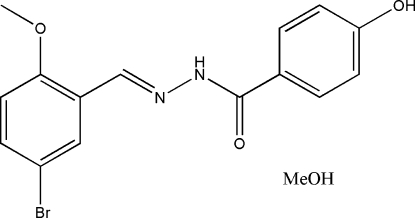


## Experimental

### 

#### Crystal data

C_15_H_13_BrN_2_O_3_·CH_4_O
*M* *_r_* = 381.23
Orthorhombic, 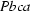

*a* = 11.1886 (7) Å
*b* = 14.4464 (9) Å
*c* = 20.5927 (13) Å
*V* = 3328.5 (4) Å^3^
*Z* = 8
Mo *K*α radiation
μ = 2.49 mm^−1^
*T* = 298 K
0.23 × 0.20 × 0.20 mm

#### Data collection

Bruker SMART CCD area-detector diffractometer
Absorption correction: multi-scan (*SADABS*; Sheldrick, 1996 ▶) *T*_min_ = 0.598, *T*_max_ = 0.636
19306 measured reflections
3638 independent reflections
2234 reflections with *I* > 2σ(*I*)
*R*_int_ = 0.054

#### Refinement

*R*[*F* ^2^ > 2σ(*F* ^2^)] = 0.054
*wR*(*F* ^2^) = 0.179
*S* = 1.06
3638 reflections
214 parameters
1 restraint
H atoms treated by a mixture of independent and constrained refinement
Δρ_max_ = 0.95 e Å^−3^
Δρ_min_ = −0.83 e Å^−3^

### 

Data collection: *SMART* (Bruker, 2002 ▶); cell refinement: *SAINT* (Bruker, 2002 ▶); data reduction: *SAINT*; program(s) used to solve structure: *SHELXS97* (Sheldrick, 2008 ▶); program(s) used to refine structure: *SHELXL97* (Sheldrick, 2008 ▶); molecular graphics: *SHELXTL* (Sheldrick, 2008 ▶); software used to prepare material for publication: *SHELXL97*.

## Supplementary Material

Crystal structure: contains datablocks global, I. DOI: 10.1107/S1600536809022983/at2817sup1.cif

Structure factors: contains datablocks I. DOI: 10.1107/S1600536809022983/at2817Isup2.hkl

Additional supplementary materials:  crystallographic information; 3D view; checkCIF report

## Figures and Tables

**Table 1:** Hydrogen-bond geometry (Å, °)

*D*—H⋯*A*	*D*—H	H⋯*A*	*D*⋯*A*	*D*—H⋯*A*
N2—H2⋯O3^i^	0.89 (4)	2.16 (5)	3.009 (4)	158 (5)
O4—H4⋯N1	0.82	2.64	3.239 (5)	131
O4—H4⋯O2	0.82	1.96	2.729 (4)	157
O3—H3⋯O4^ii^	0.82	1.78	2.602 (5)	175

Symmetry codes: (i) 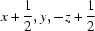; (ii) 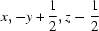.

**Fig. 1.**
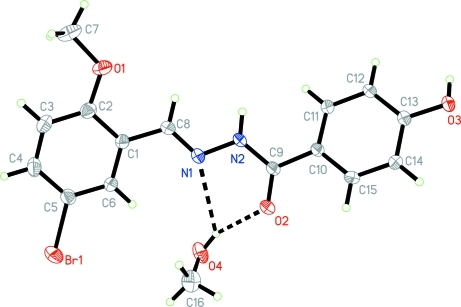
The molecular structure of (I), showing 30% probability displacement ellipsoids and the atom-numbering scheme. Intramolecular hydrogen bonds are shown as dashed lines.

**Fig. 2.**
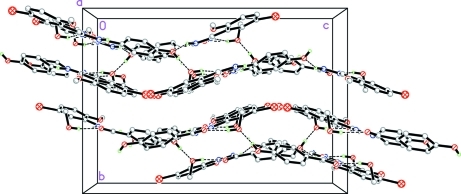
The crystal packing of (I). Hydrogen atoms not involved in hydrogen bonding have been omitted. Hydrogen bonds are shown as dashed lines.

## supplementary crystallographic information

### Comment

Hydrazone and Schiff base compounds derived from the reaction of aldehydes with hydrazides have been widely investigated both for their crystal structures and biological properties (Khattab *et al.*, 2005; Küçükgüzel *et al.*, 2003; Çukurovalı *et al.*, 2006). In the last few years, a large number of hydrazone derivatives have been reported (Fun *et al.*, 2008; Wei *et al.*, 2009; Khaledi *et al.*, 2008; Yang *et al.*, 2008). However, the hydrazone compounds derived the 5-bromo-2-methoxybenzaldehyde have never been reported. In this paper, the crystal structure of the title new hydrazone compound, (I), derived from the reaction of 5-bromo-2-methoxybenzaldehyde and 4-hydroxybenzohydrazide, is reported.

The molecular structure of (I) is shown as Fig. 1. The compound consists of a hydrazone molecule and a methanol molecule of crystallization. The methanol molecule is linked to the hydrazone molecule through intramolecular O–H···N and O–H···O hydrogen bonds, Table 1. The hydrazone molecule adopts an *E* configuration about the C═N double bond. The molecule is twisted, with the dihedral angle between the C1—C6 and C10—C15 benzene rings of 35.7 (2)°. All the bond lengths are within normal values (Allen *et al.*, 1987).

In the crystal structure of the compound, molecules are linked through intermolecular N–H···O and O–H···O hydrogen bonds, Table 1, forming layers parallel to the *ac* plane, as shown in Fig. 2.

### Experimental

5-Bromo-2-methoxybenzaldehyde (1.0 mmol, 215.0 mg) and 4-hydroxybenzohydrazide (1.0 mmol, 152.2 mg) were mixed and refluxed in methanol (50 ml). The mixture was stirred for 1 h to give a clear colourless solution. Colourless crystals of (I) were formed by slow evaporation of the solution in air for a few days.

### Refinement

H2 attached to N2 was located in a difference map and refined with N–H distance restraint of 0.90 (1) Å. The other H atoms were positioned geometrically [*d*(C–H) = 0.93–0.96 Å, d(O–H) = 0.82 Å], and refined using a riding model, with *U*_iso_(H) = 1.2*U*_eq_(C) and 1.5*U*_eq_(O and C_methyl_).

### Crystal data

**Table:**

C_15_H_13_BrN_2_O_3_·CH_4_O	*F*(000) = 1552
*M**_r_* = 381.23	*D*_x_ = 1.522 Mg m^−^^3^
Orthorhombic, *P**b**c**a*	Mo *K*α radiation, λ = 0.71073 Å
Hall symbol: -P 2ac 2ab	Cell parameters from 3350 reflections
*a* = 11.1886 (7) Å	θ = 2.5–24.5°
*b* = 14.4464 (9) Å	µ = 2.49 mm^−^^1^
*c* = 20.5927 (13) Å	*T* = 298 K
*V* = 3328.5 (4) Å^3^	Block, colourless
*Z* = 8	0.23 × 0.20 × 0.20 mm

### Data collection

**Table:**

Bruker SMART CCD area-detector diffractometer	3638 independent reflections
Radiation source: fine-focus sealed tube	2234 reflections with *I* > 2σ(*I*)
graphite	*R*_int_ = 0.054
ω scans	θ_max_ = 27.0°, θ_min_ = 2.0°
Absorption correction: multi-scan (*SADABS*; Sheldrick, 1996)	*h* = −14→14
*T*_min_ = 0.598, *T*_max_ = 0.636	*k* = −18→15
19306 measured reflections	*l* = −23→26

### Refinement

**Table:**

Refinement on *F*^2^	Primary atom site location: structure-invariant direct methods
Least-squares matrix: full	Secondary atom site location: difference Fourier map
*R*[*F*^2^ > 2σ(*F*^2^)] = 0.054	Hydrogen site location: inferred from neighbouring sites
*wR*(*F*^2^) = 0.179	H atoms treated by a mixture of independent and constrained refinement
*S* = 1.06	*w* = 1/[σ^2^(*F*_o_^2^) + (0.0849*P*)^2^ + 4.4269*P*] where *P* = (*F*_o_^2^ + 2*F*_c_^2^)/3
3638 reflections	(Δ/σ)_max_ < 0.001
214 parameters	Δρ_max_ = 0.95 e Å^−^^3^
1 restraint	Δρ_min_ = −0.82 e Å^−^^3^

### Special details

**Table:**

Geometry. All e.s.d.'s (except the e.s.d. in the dihedral angle between two l.s. planes) are estimated using the full covariance matrix. The cell e.s.d.'s are taken into account individually in the estimation of e.s.d.'s in distances, angles and torsion angles; correlations between e.s.d.'s in cell parameters are only used when they are defined by crystal symmetry. An approximate (isotropic) treatment of cell e.s.d.'s is used for estimating e.s.d.'s involving l.s. planes.
Refinement. Refinement of *F*^2^ against ALL reflections. The weighted *R*-factor *wR* and goodness of fit *S* are based on *F*^2^, conventional *R*-factors *R* are based on *F*, with *F* set to zero for negative *F*^2^. The threshold expression of *F*^2^ > σ(*F*^2^) is used only for calculating *R*-factors(gt) *etc*. and is not relevant to the choice of reflections for refinement. *R*-factors based on *F*^2^ are statistically about twice as large as those based on *F*, and *R*- factors based on ALL data will be even larger.

### Fractional atomic coordinates and isotropic or equivalent isotropic displacement parameters (Å^2^)

**Table:**

	*x*	*y*	*z*	*U*_iso_*/*U*_eq_	
Br1	0.82955 (5)	0.03436 (5)	0.73273 (2)	0.0699 (3)	
N1	0.7667 (3)	0.1520 (3)	0.48426 (15)	0.0388 (8)	
N2	0.7226 (3)	0.1789 (3)	0.42463 (16)	0.0396 (8)	
O1	1.1184 (3)	0.1179 (3)	0.49400 (17)	0.0591 (9)	
O2	0.5326 (3)	0.1584 (2)	0.45803 (13)	0.0472 (8)	
O3	0.4288 (3)	0.2326 (3)	0.16133 (13)	0.0499 (9)	
H3	0.4694	0.2697	0.1408	0.075*	
O4	0.5525 (4)	0.1537 (3)	0.59007 (15)	0.0589 (10)	
H4	0.5657	0.1611	0.5512	0.088*	
C1	0.9352 (4)	0.1136 (3)	0.54842 (19)	0.0365 (9)	
C2	1.0590 (4)	0.0944 (3)	0.5493 (2)	0.0422 (10)	
C3	1.1097 (4)	0.0555 (3)	0.6035 (2)	0.0488 (12)	
H3A	1.1908	0.0415	0.6034	0.059*	
C4	1.0431 (5)	0.0369 (4)	0.6572 (2)	0.0521 (12)	
H4A	1.0784	0.0104	0.6936	0.062*	
C5	0.9219 (4)	0.0579 (3)	0.6571 (2)	0.0412 (10)	
C6	0.8690 (4)	0.0949 (3)	0.6038 (2)	0.0381 (10)	
H6	0.7876	0.1078	0.6044	0.046*	
C7	1.2442 (5)	0.1054 (5)	0.4918 (3)	0.0724 (17)	
H7A	1.2804	0.1366	0.5279	0.109*	
H7B	1.2748	0.1307	0.4520	0.109*	
H7C	1.2625	0.0406	0.4939	0.109*	
C8	0.8794 (4)	0.1474 (3)	0.48878 (19)	0.0387 (10)	
H8	0.9269	0.1655	0.4540	0.046*	
C9	0.6031 (4)	0.1770 (3)	0.41462 (18)	0.0348 (9)	
C10	0.5622 (3)	0.1963 (3)	0.34754 (18)	0.0324 (9)	
C11	0.6285 (4)	0.2436 (3)	0.30120 (19)	0.0366 (9)	
H11	0.7031	0.2670	0.3123	0.044*	
C12	0.5861 (4)	0.2567 (3)	0.23906 (18)	0.0400 (10)	
H12	0.6320	0.2882	0.2086	0.048*	
C13	0.4747 (4)	0.2226 (3)	0.22221 (19)	0.0373 (10)	
C14	0.4067 (4)	0.1763 (3)	0.26778 (19)	0.0408 (10)	
H14	0.3320	0.1531	0.2566	0.049*	
C15	0.4499 (4)	0.1648 (3)	0.3298 (2)	0.0397 (10)	
H15	0.4026	0.1351	0.3606	0.048*	
H2	0.777 (4)	0.182 (4)	0.393 (2)	0.080*	
C16	0.5253 (6)	0.0617 (4)	0.6017 (3)	0.0657 (15)	
H16A	0.5874	0.0231	0.5844	0.099*	
H16B	0.4508	0.0466	0.5811	0.099*	
H16C	0.5187	0.0516	0.6476	0.099*	

### Atomic displacement parameters (Å^2^)

**Table:**

	*U*^11^	*U*^22^	*U*^33^	*U*^12^	*U*^13^	*U*^23^
Br1	0.0692 (4)	0.1010 (5)	0.0395 (3)	−0.0096 (3)	0.0019 (2)	0.0223 (3)
N1	0.039 (2)	0.051 (2)	0.0270 (17)	−0.0015 (17)	−0.0030 (14)	0.0055 (15)
N2	0.037 (2)	0.057 (2)	0.0242 (17)	0.0008 (18)	0.0011 (14)	0.0086 (16)
O1	0.0380 (18)	0.086 (3)	0.053 (2)	0.0017 (18)	0.0135 (15)	0.0008 (18)
O2	0.0429 (17)	0.071 (2)	0.0277 (15)	−0.0026 (16)	0.0035 (13)	0.0088 (14)
O3	0.0448 (18)	0.077 (2)	0.0283 (15)	−0.0177 (17)	−0.0081 (13)	0.0087 (15)
O4	0.080 (3)	0.067 (2)	0.0300 (16)	0.007 (2)	0.0109 (16)	−0.0003 (15)
C1	0.032 (2)	0.045 (3)	0.032 (2)	−0.0008 (19)	−0.0022 (17)	−0.0001 (18)
C2	0.036 (2)	0.048 (3)	0.042 (2)	0.002 (2)	0.0020 (19)	−0.006 (2)
C3	0.033 (2)	0.057 (3)	0.056 (3)	0.005 (2)	−0.006 (2)	−0.003 (2)
C4	0.049 (3)	0.059 (3)	0.048 (3)	0.005 (2)	−0.014 (2)	0.008 (2)
C5	0.044 (3)	0.043 (3)	0.037 (2)	−0.006 (2)	−0.0039 (19)	0.0031 (19)
C6	0.031 (2)	0.048 (3)	0.035 (2)	−0.0042 (19)	−0.0032 (17)	0.0042 (19)
C7	0.040 (3)	0.086 (4)	0.091 (4)	0.002 (3)	0.022 (3)	−0.005 (4)
C8	0.037 (2)	0.049 (3)	0.030 (2)	−0.002 (2)	0.0032 (17)	0.0052 (18)
C9	0.037 (2)	0.041 (2)	0.0264 (19)	0.0014 (19)	0.0022 (16)	0.0027 (17)
C10	0.032 (2)	0.039 (2)	0.0266 (19)	0.0022 (18)	0.0013 (16)	0.0025 (16)
C11	0.032 (2)	0.049 (3)	0.029 (2)	−0.0019 (19)	−0.0015 (16)	0.0012 (19)
C12	0.036 (2)	0.058 (3)	0.026 (2)	−0.008 (2)	0.0002 (17)	0.0042 (18)
C13	0.037 (2)	0.048 (3)	0.0275 (19)	−0.0042 (19)	−0.0018 (17)	0.0002 (18)
C14	0.031 (2)	0.054 (3)	0.037 (2)	−0.007 (2)	−0.0029 (18)	0.007 (2)
C15	0.035 (2)	0.051 (3)	0.033 (2)	−0.003 (2)	0.0062 (17)	0.0095 (19)
C16	0.071 (4)	0.059 (4)	0.067 (4)	0.004 (3)	0.010 (3)	0.006 (3)

### Geometric parameters (Å, °)

**Table:**

Br1—C5	1.899 (4)	C5—C6	1.357 (6)
N1—C8	1.266 (6)	C6—H6	0.9300
N1—N2	1.379 (4)	C7—H7A	0.9600
N2—C9	1.354 (6)	C7—H7B	0.9600
N2—H2	0.89 (4)	C7—H7C	0.9600
O1—C2	1.362 (5)	C8—H8	0.9300
O1—C7	1.420 (6)	C9—C10	1.481 (5)
O2—C9	1.222 (5)	C10—C15	1.385 (6)
O3—C13	1.363 (5)	C10—C11	1.388 (6)
O3—H3	0.8200	C11—C12	1.378 (5)
O4—C16	1.385 (6)	C11—H11	0.9300
O4—H4	0.8200	C12—C13	1.384 (6)
C1—C6	1.386 (6)	C12—H12	0.9300
C1—C2	1.413 (6)	C13—C14	1.381 (6)
C1—C8	1.462 (6)	C14—C15	1.377 (6)
C2—C3	1.371 (6)	C14—H14	0.9300
C3—C4	1.361 (7)	C15—H15	0.9300
C3—H3A	0.9300	C16—H16A	0.9600
C4—C5	1.389 (7)	C16—H16B	0.9600
C4—H4A	0.9300	C16—H16C	0.9600
			
C8—N1—N2	115.9 (3)	N1—C8—C1	120.3 (4)
C9—N2—N1	118.9 (3)	N1—C8—H8	119.8
C9—N2—H2	124 (4)	C1—C8—H8	119.8
N1—N2—H2	115 (4)	O2—C9—N2	122.0 (4)
C2—O1—C7	118.6 (4)	O2—C9—C10	121.7 (4)
C13—O3—H3	109.5	N2—C9—C10	116.3 (3)
C16—O4—H4	109.5	C15—C10—C11	117.7 (4)
C6—C1—C2	118.3 (4)	C15—C10—C9	117.6 (3)
C6—C1—C8	121.9 (4)	C11—C10—C9	124.7 (4)
C2—C1—C8	119.7 (4)	C12—C11—C10	121.5 (4)
O1—C2—C3	125.5 (4)	C12—C11—H11	119.3
O1—C2—C1	114.7 (4)	C10—C11—H11	119.3
C3—C2—C1	119.8 (4)	C11—C12—C13	119.6 (4)
C4—C3—C2	121.0 (4)	C11—C12—H12	120.2
C4—C3—H3A	119.5	C13—C12—H12	120.2
C2—C3—H3A	119.5	O3—C13—C14	117.9 (4)
C3—C4—C5	119.4 (4)	O3—C13—C12	122.2 (4)
C3—C4—H4A	120.3	C14—C13—C12	119.9 (4)
C5—C4—H4A	120.3	C15—C14—C13	119.7 (4)
C6—C5—C4	120.8 (4)	C15—C14—H14	120.1
C6—C5—Br1	119.8 (3)	C13—C14—H14	120.1
C4—C5—Br1	119.4 (3)	C14—C15—C10	121.5 (4)
C5—C6—C1	120.6 (4)	C14—C15—H15	119.2
C5—C6—H6	119.7	C10—C15—H15	119.2
C1—C6—H6	119.7	O4—C16—H16A	109.5
O1—C7—H7A	109.5	O4—C16—H16B	109.5
O1—C7—H7B	109.5	H16A—C16—H16B	109.5
H7A—C7—H7B	109.5	O4—C16—H16C	109.5
O1—C7—H7C	109.5	H16A—C16—H16C	109.5
H7A—C7—H7C	109.5	H16B—C16—H16C	109.5
H7B—C7—H7C	109.5		

### Hydrogen-bond geometry (Å, °)

**Table:**

*D*—H···*A*	*D*—H	H···*A*	*D*···*A*	*D*—H···*A*
N2—H2···O3^i^	0.89 (4)	2.16 (5)	3.009 (4)	158 (5)
O4—H4···N1	0.82	2.64	3.239 (5)	131
O4—H4···O2	0.82	1.96	2.729 (4)	157
O3—H3···O4^ii^	0.82	1.78	2.602 (5)	175

Symmetry codes: (i) *x*+1/2, *y*, −*z*+1/2; (ii) *x*, −*y*+1/2, *z*−1/2.
